# Innovative strategies for sustainable food processing: enhancing quality, reducing waste, and promoting circular economy^☆^

**DOI:** 10.1016/j.fochx.2025.103445

**Published:** 2026-01-28

**Authors:** Vinod Kumar, Narpinder Singh, Katsuyoshi Nishinari

**Affiliations:** a Department of Food Science and Technology, Graphic Era (Deemed to be University), Dehradun 248002, India; b Hubei University of Technology, Wuhan, Hubei Province, PR China, 430068

Nowadays, sustainable food processing has become an important research area because conventional food processing techniques contribute to food wastage and environment depletion. Wang et al. (2025) reported that there is need to redesign the policies to ensure global food security. There are limited studies that embed food security, circular economy and sustainable food system. The current scenario demands the adoption of sustainable food system to achieve food security. Therefore, the special issue “Innovative Strategies for Sustainable Food Processing: Enhancing Quality, Reducing Waste, and Promoting Circular Economy” integrates the application of sustainable food system and circular economy driven strategies for the future. This special issue focused on following diverse sub-themes.

## Agri-food waste reduction and circular economy approaches

This subtheme highlights efforts to reduce agri-food waste and turn it into valuable products as part of a circular economy. Pandey et al. (2025) examined agri-food waste streams and identified new ways to convert them into nutraceuticals, biofuels, fermented foods, and industrial materials. Additionally, Nautiyal et al. (2025) took this discussion forward and suggested the application of deep machine learning and AI in agricultural practices for reducing waste. The use of these advanced digital techniques can be helpful in smart irrigation and precision agriculture. Moving ahead, valorization of agri-food waste can also contribute to sustainable food system. Thapa et al. (2025) reported the use of underutilized by Sal seeds for the extraction of functional edible oil.

## Green extraction and sustainable processing technologies

This subtheme covers the development of green extraction and sustainable processing technologies that improve efficiency and lower environmental impact. Singh et al. (2025) reported the use of microwave extraction technique for extraction of bioactive compounds from *Piper betel* L with higher yield. In addition, Tan et al. (2025) used ultrasound-assisted extraction to recover bioactive antifreeze peptides from shrimp (*Litopenaeus vannamei*) processing waste, showing a practical way to turn waste into valuable products in line with circular economy ideas.

## Sustainable food ingredients and biotechnological innovations

Numerous studies have covers biotechnological and ingredient-level advancements for the development of sustainable food products. Sahal et al. (2025) focused on the nutritional and bioactive properties of nettle (*Urtica dioica*) leaves and their potential use in edible coatings. Bhtoya et al. (2025) showed how cucumber and watermelon seed powders can be utilized as functional ingredients in fortified dairy desserts, contributing to waste reduction and nutritional improvement. The study by Kamboj et al. (2025) concluded that fermented barnyard millet protein hydrolysate had antimicrobial properties, which can be utilized in nutraceutical and functional food product development.

## Environmental sustainability and allied technologies

In addition to food processing, this Special Issue covers related technologies that help support sustainable food systems. Rana et al. (2025) reported that nanocellulose membrane can be utilized for waste water treatment rather than conventional filtration system. In addition, Kothari and Kumar (2025) reported that metallic and carbon-based nanomaterials can be used for the sustainable dairy farming.

## Conclusion

Collectively, these studies have the potential to promote sustainable food system and circular economy. The value-added products, novel ingredients and technologies discussed can lead to reduce emission load on environment. By using green processing methods, finding new uses for waste, and applying digital and biotechnological tools, these papers show practical steps toward building more sustainable and resilient food systems. The research makes it clear that sustainability in food processing needs a broad approach, covering everything from how raw materials are used to processing efficiency, by-product management, and safeguard the environment.

## Future perspectives and directions

Future research must focus on bridging the gap between laboratory innovation and industrial application. While green extraction and waste valorization show promise, comprehensive Life Cycle Assessments (LCA) are essential to quantify their true long-term environmental impact. The integration of Industry 4.0, particularly AI-driven process optimization, will be vital for minimizing resource and energy wastage. Furthermore, overcoming regulatory hurdles and enhancing consumer acceptance of upcycled ingredients remains a significant priority. Ultimately, a multi-disciplinary approach combining biotechnology, material science, and policy is required to transform these waste-to-value strategies into resilient, global circular food systems that effectively ensure food security while staying within planetary boundaries.
